# Discovery and excavation of lichen bioactive natural products

**DOI:** 10.3389/fmicb.2023.1177123

**Published:** 2023-04-17

**Authors:** Meirong Ren, Shuhua Jiang, Yanyan Wang, Xinhua Pan, Feng Pan, Xinli Wei

**Affiliations:** ^1^Key Laboratory of Biodiversity Conservation in Southwest China, State Forestry Administration, Southwest Forestry University, Kunming, China; ^2^State Key Laboratory of Mycology, Institute of Microbiology, Chinese Academy of Sciences, Beijing, China; ^3^Jiangxi Xiankelai Biotechnology Co., Ltd., Jiujiang, China

**Keywords:** lichen, natural products, bioactivity, PKS, OSMAC strategy, genome mining

## Abstract

Lichen natural products are a tremendous source of new bioactive chemical entities for drug discovery. The ability to survive in harsh conditions can be directly correlated with the production of some unique lichen metabolites. Despite the potential applications, these unique metabolites have been underutilized by pharmaceutical and agrochemical industries due to their slow growth, low biomass availability, and technical challenges involved in their artificial cultivation. At the same time, DNA sequence data have revealed that the number of encoded biosynthetic gene clusters in a lichen is much higher than in natural products, and the majority of them are silent or poorly expressed. To meet these challenges, the one strain many compounds (OSMAC) strategy, as a comprehensive and powerful tool, has been developed to stimulate the activation of silent or cryptic biosynthetic gene clusters and exploit interesting lichen compounds for industrial applications. Furthermore, the development of molecular network techniques, modern bioinformatics, and genetic tools is opening up a new opportunity for the mining, modification, and production of lichen metabolites, rather than merely using traditional separation and purification techniques to obtain small amounts of chemical compounds. Heterologous expressed lichen-derived biosynthetic gene clusters in a cultivatable host offer a promising means for a sustainable supply of specialized metabolites. In this review, we summarized the known lichen bioactive metabolites and highlighted the application of OSMAC, molecular network, and genome mining-based strategies in lichen-forming fungi for the discovery of new cryptic lichen compounds.

## Introduction

Plant-derived natural products or their derivatives were a valuable source of therapeutic agents and played a key role in the treatment of various diseases, e.g., cancer chemotherapy, infectious diseases, cardiovascular diseases, and lipid metabolism disorders (Mann, 2002; Newman and Cragg, 2012; Atanasov et al., 2015; Waltenberger et al., 2016). However, natural plant product-based drug discovery has some difficulties because of technical barriers to screen natural products in high-throughput assays against molecular targets and synthetic compounds not meeting the expectations of an increasing number of new drugs reaching the market (Atanasov et al., 2015), thus scientists have had to turn their attention to other organisms. Microbes have proven to be a bountiful source of secondary metabolites that have been successfully developed as crucial drug leads. We have already known that the structures of over 80,000 natural products from microbes (Demain, 2014) and over 80% of the antibiotics are produced by microbes (de Lima Procópio et al., 2012) since the discovery of penicillin in 1928 (Demain, 2014). Due to the extensive use of antibiotics for common infections, pathogens are showing high resistance (Harikumar and Krishanan, 2022); therefore, there is an urgent need for finding novel drugs. Grube made a point of view that microbial symbioses have a high potential leading to a wide variety of unique structures and metabolic activities (Grube and Berg, 2009).

**Figure 1:**
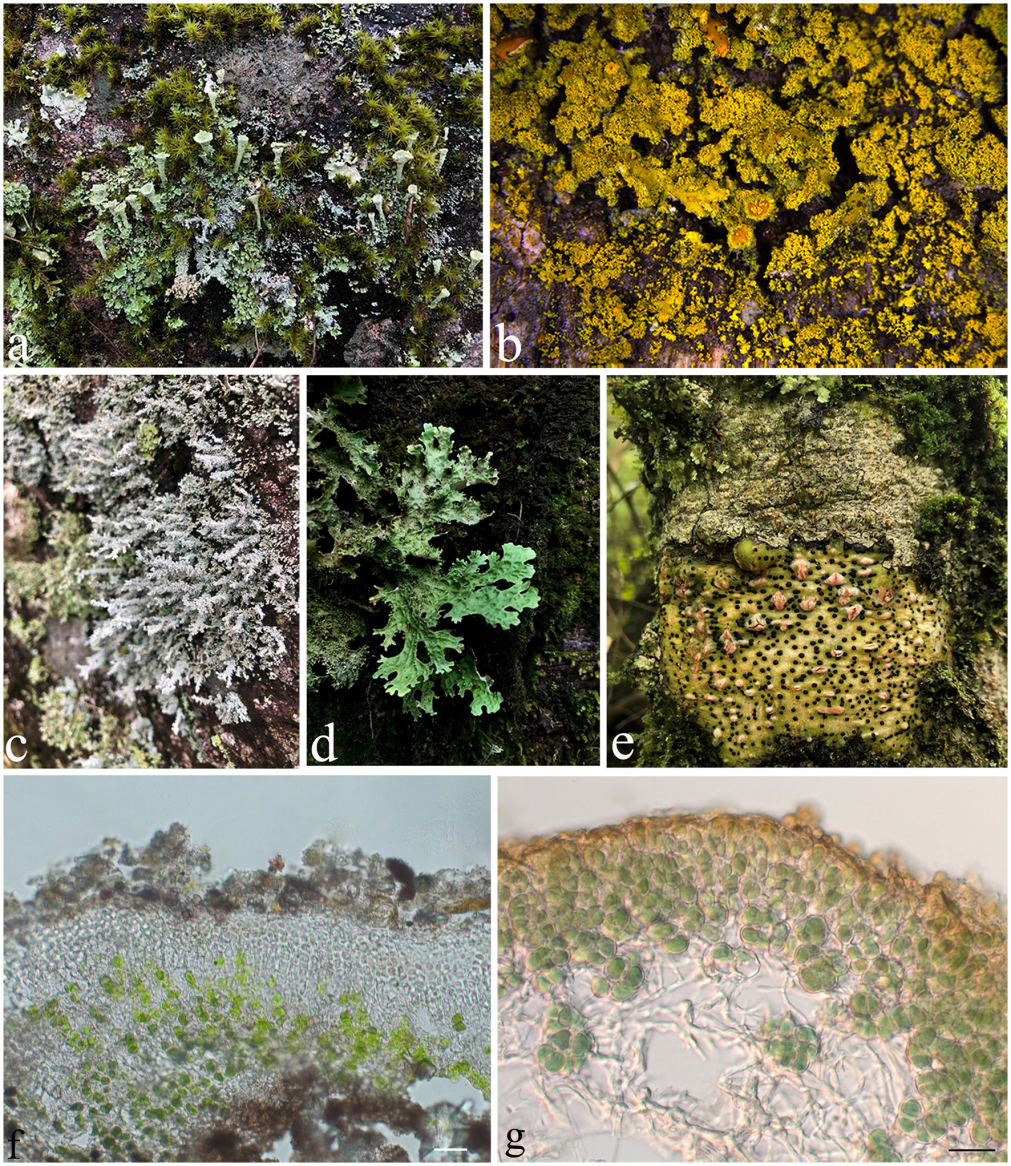
The diversity of lichens. **(a)** *Cladonia* sp.; **(b)** *Candelaria* sp.; **(c)** *Stereocaulon* sp.; **(d)** *Sticta* sp.; **(e)** *Pyrenula* sp.; **(f)** Thallus of *Endocarpon pallidulum* in vertical section, with green algae *Diplosphaera chodatii* as the photobiont; **(g)** Thallus of *Peltula submarginata* in vertical section, with cyanobacteria Chroococcales as the photobiont. Photos **(b, f)** were taken by Xue XD and Zhang TT, respectively, and photos **(a, c–e)** were taken by Yang QX. Scale bars: **(f)** = 20 μm, **(g)** = 10 μm.

Several studies have shown that lichens are productive organisms for the synthesis of a broad range of secondary metabolites. Lichen is a stable community in the ecosystem of the Earth's biosphere, which is composed of a mutualistic relationship between fungi and algae or between fungi and cyanobacteria (Figure 1). However, the identity of lichens is considered based on the fungal partner, and to date, the predominant records of lichens that have been identified are ascomycetes in nature. Thus, the term “lichen-forming fungi (LFF)” refer to the fungi that live in lichen thallus throughout the entire life cycle by establishing a co-benefit symbiotic relationship without causing any adverse effect and are different from those endolichenic fungi (Muggia et al., 2009).

Evidence from lichen fossils indicated that the interactions between fungi and algae have existed for at least 400 million years (Lücking and Nelsen, 2018), and studies have shown that lichen occurs over 10% of the terrestrial surface, especially extreme and aggressive environmental conditions that are not conducive to individual survival, such as extreme cold Arctic and Antarctic regions (Lee et al., 2014), hot and arid deserts (Kranner et al., 2008), alpine areas with strong UV irradiation, and on rocks or non-fertile soils (de Vera et al., 2003; Seymour et al., 2005; Boustie et al., 2010; Nguyen et al., 2013). This tendency of lichens to tolerate the extreme environment can be correlated with the production of both a unique and diverse range of metabolites known as lichen substances (Schweiger et al., 2022). Fungi and algae co-evolved unique biosynthetic pathways and metabolic mechanisms to synthesize these complex metabolites over a long period of time. Primary and secondary metabolisms are the two main groups of lichen compounds. Primary metabolism is the basic substance constituting the structure of lichen and includes proteins, amino acids, carotenoids, polysaccharides, and vitamins (Goga et al., 2020; Packiam and Perumal, 2022). The fungal filaments provide small, structurally complex, water-insoluble, and crystalline secondary metabolism, which can comprise up to 20% of the dry mass of thallus weight (Nguyen et al., 2013; González-Burgos et al., 2019; Zhao et al., 2021). Unlike primary metabolites, lichen secondary metabolism is not directly involved in growth but synthesized for algae or cyanobacteria protection (Muggia et al., 2009).

**Figure 2:**
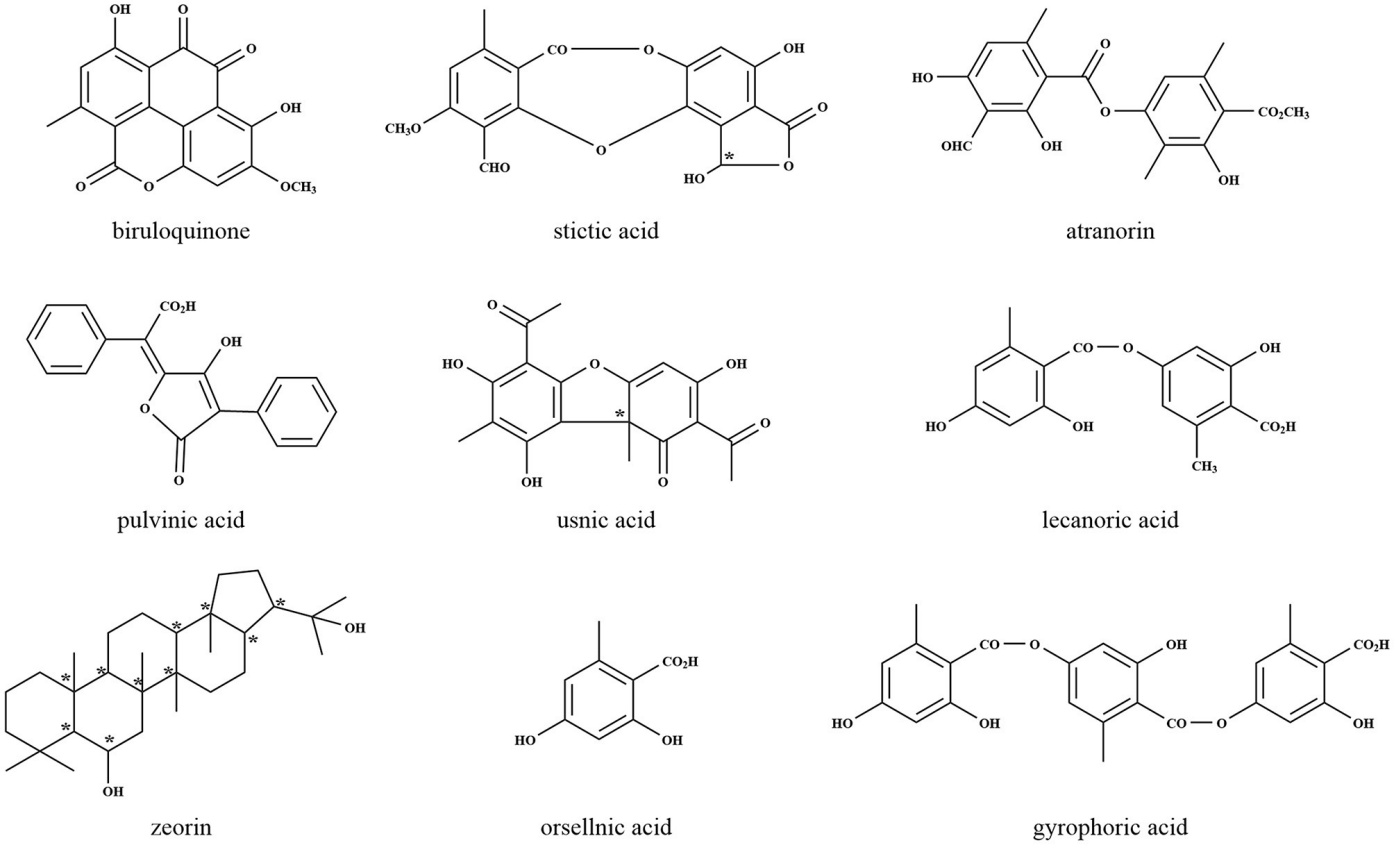
Representative lichen metabolites derived by LFF. *Indicates the chiral centers.

Lichens are known to produce small molecular compounds such as aliphatic and aromatic compounds, thus far, over 1,000 compounds have been identified (Shrestha and St. Clair, 2013). According to biosynthetic origins and chemical structures, lichen compounds were classified (Culberson and Elix, 1989), which were synthesized by acetyl-malonate, mevalonate, and shikimate pathways existing in all organisms as key routes for natural metabolism. The biosynthesis of lichen depsides, depsidones, dibenzofurans, chromones, xanthones, and anthraquinones occurs via the acetyl-malonate pathway, by which most bioactive compounds are synthesized, with coenzyme A as the precursor and polyketide synthase (PKS) as the responsible enzyme (Ibrahim et al., 2018). The most common lichen compounds synthesized by this pathway include evernic acid (Muggia et al., 2009), lecanoric acid (Lawrey, 1986), gyrophoric acid (Garima et al., 2022a), atranorin (Lawrey, 1986; Majchrzak-Celinska et al., 2022), thamnolic acid (Culberson et al., 1986; Jeong et al., 2021), umbilicaric acid (Posner et al., 1991; Yoshimura et al., 1994), protocetraric acid (Nishanth et al., 2015), fumarprotocetraric acid (Igoli et al., 2014; Ranković and Mišić, 2014), stictic acid (Bellio et al., 2017; Pejin et al., 2017), usnic acid (Moreira et al., 2015; Sepahvand et al., 2021), lepraric acid (Aberhart et al., 1969; Murugan et al., 2021), and thiophanic acid (Arshad et al., 1968; Dayan and Romagni, 2001). Usnic acid, one of the most common, isolated, and discussed lichen compounds, is well-known as an antibiotic with many pharmacological activities including antibacterial, antiprotozoal, anti-cytotoxic, anti-proliferative, antioxidant, and anti-inflammatory (Cocchietto et al., 2002). The mechanisms of bioactivity of usnic acid modify the structures of proteins causing irreversible changes and may even produce apoptosis. Lichen also produces an array of secondary metabolites derived from the mevalonate pathway, which play essential roles in the regulation of cell growth and development, and the products appear to be potentially interesting therapeutic targets for many areas of research such as oncology, autoimmune disorder, atherosclerosis, and Alzheimer disease (Buhaescu and Izzedine, 2007). The mevalonate pathway is mainly associated with the production of terpenes, steroids, and carotenoids (Goga et al., 2020). Until now, more than 20 different triterpene compounds from lichens have been reported. The shikimic acid pathway, ubiquitous in microorganisms and plants, provides precursors for the biosynthesis of primary metabolites such as aromatic amino acids and folic acid (Wilson et al., 1998). This pathway is mainly related to pulvinic acid and terphenylquinone pigments (Edwards et al., 2003), which help lichen adapt to UV stress by absorption and re-emitting the UV radiation as fluorescence or heat (Nguyen et al., 2013). The representative structures of lichen natural products are shown in Figure 2.

Lichen cells contain many types of natural metabolites and other bioactive molecules, receiving increased attention due to their industrial, pharmaceutical, biotechnological, medical, and cosmetics applications (Elkhateeb and Daba, 2019). Many studies have supported the potentiality of different lichen species to produce unique natural compounds with different physicochemical and biological activities (Hamida et al., 2021). Their utilization in folklore as medicines in the treatment of diverse diseases, such as arthritis, alopecia, constipation, kidney diseases, leprosy, pharyngitis rabies, infection, worm, and infestation for several centuries, has been recorded in different pharmacopeias by native Americans, Haitian, Indians, Chinese, and Europeans (Romagni and Dayan, 2002; Elkhateeb and Daba, 2019). In Table 1, the known lichen natural products with different bioactivities were summarized, among which most were isolated from the natural lichen thallus.

**Table 1:** The known lichen bioactive natural products from a thallus or LFF cultures.

**Class of compound**	**Natural compound**	**Bioactivity**	**Lichen species**	**References**
Aliphatic and cycloaliphatic compound	D-protolichesterinic acid/nephrosterinic acid	Anticancer	*Ramalina almquistii/Usnea longissima*	Bessadottir et al., 2015; Reddy et al., 2019
	Protolichesterinic acid	Anti-bacterial/inflammatory proliferation/antimicrobial/anticancer	*Hypotrachyna cirrhata/Cornicularia aculeata/Cetraria islandica*	Bessadottir et al., 2014; Furmanek et al., 2021
	Roccellic acid	Antimicrobial	*Roccella montagnei*	Mishra et al., 2017
Anthraquinones	Emodin	Antifungal	*Xanthoria parietina/Caloplaca aurantia/Nephroma laevigatum*	Manojlovic et al., 1998
	Fallacinal	Antifungal	*Xanthoria parietina*	Manojlovic et al., 1998
	Parietin	Cytotoxicity/antimicrobial	*Xanthoria parietina*	Pichler et al., 2021
Depside	Nordivaricatic acid	Human leukocyte elastase inhibitor	Parmelia saxatilis^*^	Zheng et al., 2012; Díaz et al., 2020
	Atranorin and derivatives	Anti-hepatitis C virus/anticancer/antimicrobial	*Stereocaulon evolutum/Cladonia rangiferina/Parmotrema austrosinense*	Lawrey, 1986; Kumar et al., 2018; Tekiela et al., 2021; Majchrzak-Celinska et al., 2022
	Baeomycesic acid	Anticancer	*Thamnolia vermicularis* var *subuliformis/T. subuliformis*	Ingolfsdottir et al., 1997
	Chloroatranorin	Antimicrobial	*Pseudevernia furfuracea/Hypotrachyna cirrhata/Parmotrema austrosinense*	Türk et al., 2006; Kumar et al., 2018; Furmanek et al., 2021
	Erythrin	Antioxidant	*Parmotrema grayana*	Thadhani et al., 2011
	Evernic acid	Antibiotics/antioxidant/cytotoxic	*Evernia prunastri/E. divaricata/Pseudoevernia furfuraceae/Roccella montagnei*	Lawrey, 1986; Kosanic et al., 2013; Mishra et al., 2017
	Isidiphorin	Antioxidant	*Usnea longissima/Lobaria pulmonaria*	Atalay et al., 2011
	Isodivaricatic acid	Antifungal/antiprotozoal	*Protousnea poeppigii*	Schmeda-Hirschmann et al., 2008
	Lecanoric acid	Anticancer/antioxidant/inhibitor of histidine decarboxylase/antifungal	Umbilicaria antarctica/Parmotrema tinctorum/Roccella montagnei/Parmelina tiliacea^*^	Umezawa et al., 1974; Lopes et al., 2008; Luo et al., 2009; Tatipamula et al., 2019; Díaz et al., 2020; Majchrzak-Celinska et al., 2022
	Lepraric acid	Antibacterial	*Roccella phycopsis*	Parrot et al., 2015
	Olivertoric acid	Antimicrobial	*Pseudevernia furfuracea*	Türk et al., 2006
	Perlatolic acid	Anti-inflammatory	*Cetrelia monachorum*	Oettl et al., 2013
	Squamatic acid	Anticancer	*Cladonia. unclalis*	Majchrzak-Celinska et al., 2022
Depsidone	α-Collatolica acid	Antimicrobial	Lecanora atra/Arctoparmelia centrifuga^*^	Neeraj et al., 2011; Pierangelo et al., 2015; Bellio et al., 2017; Díaz et al., 2020
	Lobaric acid	Cytotixic/anti-inflammatory/antimicrobial/enzyme inhibition/muscle relaxant/antioxidant/	*Stereocaulon alpinum/S. paschale/Usnea longissima/Cladonia* sp./Parmelia saxatilis^*^	Gissurarson et al., 1997; Seo et al., 2009; Thadhani et al., 2011; Pierangelo et al., 2015; Joo et al., 2016; Kwon et al., 2016; Bellio et al., 2017; Claudia et al., 2018; Hong et al., 2018; Kim, 2018; Emsen et al., 2019; Lee et al., 2019; Díaz et al., 2020
	Norstictic acid	Antimicrobial/cytotoxic/antioxidant	Toninia candida/Ramalina farinacea/Stereocaulon montagneanum/Usnea strigose/Xanthoparmelia tinctina^*^	Tay et al., 2004; Rankovic et al., 2012; Ebrahim et al., 2016; Ismed et al., 2017; Díaz et al., 2020
	Stictic acid	Cytotoxic/antibiotics/antioxidant	*Lobaria pulmonaria/Rhizoplaca aspidophora/Xanthoparmelia camtschadalis/S. montagneanum/Hypotrochyna revolute/Usnea longissima*	Papadopoulou et al., 2007; Atalay et al., 2011; McGillick et al., 2016; Bellio et al., 2017; Ismed et al., 2017; Pejin et al., 2017
	Fumarprotocetrari acid	Antimicrobial/antioxidant expectorant/photoprotection	*Cladonia foliacea/Cetraria islandica/Cladonia verticillaris/Lasallia pustulata/Evernia prunastri*	Lawrey, 1986; Dévéhat et al., 2013; de Barros Alves et al., 2014; Igoli et al., 2014; Ranković and Mišić, 2014; Tekiela et al., 2021
	Physodic acid	Anticancer/antioxidant/antimicrobial	*Hypogymnia physodes/Evernia prunastri/Pseudevernia furfuracea*	Kosanic et al., 2013; Majchrzak-Celinska et al., 2022
	Salazinic acid	Anti-Alzheimer/antioxidant/anticancer/antibacteria	*Parmelia sulcata/P. saxatilis/Everniastrum cirrhatum/Rimelia cetrata/Leucodermia leucomelos/Xanthoparmelia camtschadalis*	Manojlovic et al., 1998; Gaikwad et al., 2014; Bellio et al., 2017; Paluszczak et al., 2018; Furmanek et al., 2021; Majchrzak-Celinska et al., 2022
	α/β-Alectoronic acid	Antimicrobial/anticancer	Alectoria sarmentosa*/Parmelia tiliacea*/Xanthoparmelia tinctina^*^/Arctoparmelia centrifuga^*^	Gollapudi et al., 1994; Díaz et al., 2020
	Physodic acid	Anticancer/antioxidant/antimicrobial	Parmelia saxatilis^*^	Díaz et al., 2020
	Neuropogonines A-C	Antimicrobial	*Neuropogon* sp.	Ivanova et al., 2002
	Pannarin	Anticancer	*Psoroma reticulatum*	Russo et al., 2006, 2008
	Pseudodepsidones 1 and 2	Anti-diabetes	*Stereocaulon alpinum*	Seo et al., 2009
	Psoromic acid	Antibiotics/antivirus	*Usnea* spp./*Psoroma* spp./*Alectoria* spp.	Lawrey, 1986
	Pulmonarianin	Lipid peroxidation inhibition/antioxidant	Usnea longissima/Lobaria pulmonaria/Xanthoparmelia tinctina^*^	Atalay et al., 2011; Díaz et al., 2020
Dibenzofuranes	Usnic acid and derivatives	Antimicrobial/analgesic/antipyrietic/anti-inflammatory, anti-cancer/antimutagrnic activity/enzyme inhibitor/anti-allergies/antivirus/plant growth inhibitor	*Usnea* spp./*Ramalina* spp.*/U. longissima^*^	Lawrey, 1986; Wang et al., 2014; Moreira et al., 2015; Sepahvand et al., 2021
Didepsides	Barbatic acid	Anticancer	*Usnea longissima*	Reddy et al., 2019
	Divaricatic acid	Antimicrobial	*Evernia divaricata*	Çobanglu et al., 2010
	Diffractaic acid	Antioxidant/anti-inflammatory	*Usnea longissima/Lobaria pulmonaria*	Bayir et al., 2006; Atalay et al., 2011
	Sekikaic acid	Anticancer/antivirus	*Ramalina farinacea*	Yousuf et al., 2014
Dimeric tetrahydroxanthone	Hirtusneanoside	Antibacterial	*Usnea hirta/Ramalina farinaceae/Peltigera polydactyla*	Rezanka and Sigler, 2007
Lichenan	β-D-1,3/1,4-glucan	Wound healing	*Cetraria islandica*	Zacharski et al., 2018
Monocyclic aromatic compound	Atranol	Antimicrobial	*Roccella montagnei*	Tatipamula et al., 2019
	Orcinol	Antimicrobial	*Roccella montagnei*	Tatipamula et al., 2019
	Orsellinic acid	Antimicrobial	*Parmotrema austrosinense*	Kumar et al., 2018
Phenanthrenequinones	Biruloquinone	Achtylcholine inhibitor	Cladonia macilenta*	Luo et al., 2013
		Acetylcholinesterase inhibitor		Luo et al., 2013
Poly-carboxylic fatty acid	Caperatic acid	Anti-inflammatory/cytotoxity/central nervous system therapeutics	*Platiamatia glauca*	Majchrzak-Celinska et al., 2022; Studzinska-Sroka et al., 2022
Polysaccharides	Polysaccharides	Antioxidant/anticancer/antiviral	*Umbilicaria esculenta/Parmelia perlata*	Olaleye et al., 2007; Sun et al., 2018; Wang et al., 2018
Pulvinic acid derivatives	Vulpinic acid	Antibiotics	Letharia columbiana/L. vulpina/Pseudocyphellaria flacicans/Vulpicida pinastri*	Lawrey, 1986; Kowalski et al., 2011
Terphenylquinine	Polyporic acid	Anticancer	*Sticta coronata*	Goga et al., 2020
	Thelephoric acid	Antioxidant/anti-alzheimer	*Lobaria isidiosa*	Rao et al., 1965; Kwak et al., 1999; Chon et al., 2016
Tridepsides	Gyrophoric acid	Anticancer	*Umbilicaria* sp./U. freyi*	Burlando et al., 2009; Garima et al., 2022a
	Tenuiorin	Anticancer	*Peltigera aphthosa/Lobaria linita/Pseudocyphellaria crocata*	Ingolfsdottir et al., 2002
Triterpenoids	Zeorin	Antimicrobial	*Leucodermia leucomelos*	Furmanek et al., 2021

* Represents the lichen compounds from LFF cultures besides lichen thallus in this species.

## Strategy to discover lichen natural products

Despite the great potential of lichen bioactivity, lichens have been long neglected by mycologists and overlooked by the pharmaceutical industry. One reason is their slow growth in nature and difficult culture in either fermenters or glasshouses, or even cultivated in the open air, and has scarcely been studied from a biochemical perspective; another reason is due to difficulties in obtaining them in sufficient quantities and purities for structural elucidation and pharmacological research. These circumstances prompted lichenologists to develop more suitable strategies to look for more lichen compounds in categories and activities.

### OSMAC strategy

To improve the production of a wider range of natural products from a microorganism, different culturing conditions are generally used. Bode et al. refer to the fact that a single strain is capable of producing a diverse array of structural compounds under specific growth conditions (Zahroh et al., 2022), however, never produces the entire compounds at the same time under one set of environmental conditions because it is not matching with a cost between energetic and metabolism (Zarins-Tutt et al., 2016). Very small changes in the cultivation parameters, such as culture medium composition, pH, temperature of growth, salinity, aeration, and even the shape of culture vessel used, can completely alter, induce, or optimize the physiology of a microbial strain and in turn significantly affect the biosynthesis of such metabolites (Bode et al., 2002). For example, bioactivity-guided isolation of the fungus *Aspergillus versicolor* KU258497 yielded two new and six known cryptic metabolites when co-cultivated with the bacterium *Bacillus subtilis* 168 trpC2 on solid rice medium, among which one new compound showed strong cytotoxic activity against the mouse lymphoma cell line L5178Y (Abdelwahab et al., 2018). A corresponding strategy named OSMAC (one strain of many compounds) opened up a new industrial production avenue for compounds needed. For lichens, the majority of the bioactive compounds are exclusively produced by the LFF; however, there are some instances where the symbiotic photobiont, particularly cyanobacteria also engaged in the production of some key secondary metabolites (Cox et al., 2005).

It has been indicated that there is a strong application basis of OSMAC in discovering lichen natural products. Especially, lichenologists from all over the world are becoming more and more interested in not only physiology but also metabolite production (Crittenden and Porter, 1991), thus, they hoped that mycelium from the lichen thallus may be free-grown on artificial medium and produced lichen compounds without the algal or cyanobacterial partners. The tissue culture method invented by Yoshimura et al. (1993), a technique for the separation of the LFF from lichen thallus and culturing it alone, greatly pushed the fulfillment of this process.

However, growth rate and metabolite yield in LFF are inverse relationships (Timsina et al., 2013) and are influenced by culturing conditions, such as the availability and type of carbon and nitrogen source (Calvo et al., 2002; Behera et al., 2006; Verma et al., 2012). Simple sugar such as glucose, sucrose, and polyethylene glycol as sole carbon sources supported high LFF growth and production of usnic acid in *Usnea ghattensis* culture, in contrast, nitrogen sources such as amino acids (glycine, asparagine, alanine, or vitamins), especially glycine, supported the LFF growth but did not well-support usnic acid production (Behera et al., 2006). A strain of LFF isolated from a thallus of *Parmotrema reticulatum* was cultured on different culture media, and all the colonies developed well; however, atranorin, the major cortical lichen depside, was only detected when the colonies were grown over 5 and 10 months on solid LB medium. By comparison, colonies grown on MEYE and MY10 with a gradually dry treatment did not synthesize any lichen secondary metabolite of polyketides but primary triacylglycerides and fatty acids as the major metabolites (Fazio et al., 2009).

Bu'Lock proposed that mycelial growth was slow under conditions of poor nutrition, but secondary metabolism could be induced (Bu'Lock et al., 1974), which is related to the carbon-nutrient balance hypothesis (Bryant et al., 1983). From LFF of *Endocarpon pusillum* cultured on the optimized PDB, nine secondary metabolites including two new isoindolin-1-ones were detected, while three known compounds and a new naphthoquinone were isolated from the rice culture (Liu R. D. et al., 2022). Temperature is another important factor influencing LFF cultivation and chemical diversity in secondary metabolism. It is due to higher or lower temperatures that will inhibit the enzyme secretion of LFF (Feller et al., 1994).

Therefore, it is greatly deserved to expect more lichen natural products will be discovered by OSMAC strategy after changing and improving a series of cultural conditions. However, there is an inevitable problem existing in this process, that is, what is the relationship between more lichen products and the valuable bioactivity of these products because lichen secondary metabolism mainly originates from the fungal partner, i.e., LFF, but is produced when the organisms are in symbiotic association (Moreira et al., 2015). Poor nutrition sources and slow growth rate are the natural factors in LFF decided by the characteristics of lichen symbiosis. *Heterodea muelleri* in the field produced diffractaic and barbatic acids, whereas the LFF cultures did not contain any detectable secondary metabolites (Hager et al., 2008). The study of temperature by Hamada in LFF and lichen thallus of *Ramalina siliquosa* examined changing of polyketides, that is, the quantity of depside produced by LFF of *Ramalina siliquosa* was the highest at optimal culture conditions (Hamada, 1989), on the contrary, depsidone was increased in *R. siliquosa* thallus (Hamada, 1981). Brunauer showed that the LFF of *Xanthoria elegans* produces secondary metabolism that is not present in the naturally collected lichen thallus by HPLC examination because the presence of gene clusters enables LFF to produce a potentially larger variety of polyketides than thallus (Brunauer et al., 2007). Another example, two new dibenzofurans, isostrepsilic acid and hypostrepsilic acid, are synthesized in large quantities by LFF culture of *Umbilicaria orientalis* on malt-yeast extract medium containing sugar alcohols, but they have not been produced by this lichen in the field (Kon et al., 1997). The production of lichen compounds is based on the resistance to extreme environments, and if the environment changes, the interactions between lichen and the environment will change, similarly, LFF is out of symbiosis when they grow on the culture medium and equal to the environment change; thus, the compounds of LFF are different from lichen symbiont, and correspondingly, some lichen bioactive compounds cannot be detected in the LFF cultures (Dayan and Romagni, 2001).

However, when quantitatively and variously LFF metabolites were obtained after more focusing on fermentation broth and mycelium, they were often found different from those contained in the natural lichen thallus (Miyagawa et al., 1993), while the reason why we are interested in the lichen natural products is due to the bioactive metabolites produced by the symbiotic lichen thallus. Whether the bioactivity of more and different lichen natural products produced in the LFF by OSMAC strategy is much better than those less in the lichen thallus is not fully understood. Anyway, there would be a long way to go on well balancing this current conflict. Well understanding and solving this problem still need to establish on the more and more discovering of lichen natural products through OSMAC under breaking the lichen symbiosis, which is also closely related to the requirement of increasing the growth rate of LFF. Here, we present Table 2 to show some optimized media and cultural conditions being reported.

**Table 2:** Optimized media and culture conditions for lichen-forming fungi (LFF).

**LFF species**	**Medium**	**Culture condition**	**Note**	**References**
*Usnea ghattensis*	MY + 10 mM Sucrose + 10 mM Polyethylglycol	18°C, 8 h light (400 lux)/16 h dark and 50–80% relative humidity, 3 months	Accelerated the growth via activating the cytochrome respiratory system	Verma et al., 2011
*Haematomma sp. Graphis proserpens*	MY + 10% sucrose	−18°C in the dark for 11 months	Promote the production of new compounds	Takenaka et al., 2011; Tanahashi et al., 2017
*Endocarpon pusillum*	Optimized PDA: 2 g/L yeast extract, 2 g/L soy peptone, 40 g/L sucrose, 200 g/L boiled potato juice	19°C on a rotary shaker at 120 rpm for 100 days	Accelerated the growth	Zhang and Wei, 2011
*Usnea longissima*	MY + 2% or 10% (w/v) inositol, annitol, sorbitol, sucrose, glucose, or fructose.	Aer 2 months of culture on MY basal medium 15°C, the mycelia were transferred into optimized MY medium	Accelerated the growth	Wang et al., 2011
*Evernia divaricata*	LB + 20 ml bark extreact	–	Promote the production of polyketides compounds	Stocker-Wörgötter and Hager, 2008
*Heterodea muelleri*	LB + 20 ml soil extract	–	Promote the production of polyketides compounds	Stocker-Wörgötter and Hager, 2008
*Cryptothecia rubrocincta*	LB + 4% erythriol		Promote the production of polyketides compounds	Stocker-Wörgötter and Hager, 2008
*Cladonia furcata*	LB + 4% ribitol		Promote the production of polyketides compounds	Stocker-Wörgötter and Hager, 2008
*Bunodophoron patagonicum*	MS + 4% sucrose		Promote the production of polyketides compounds	Stocker-Wörgötter and Hager, 2008
*Stereocaulon ramulosum*	Sabouraud 4% glucose agar		Promote the production of polyketides compounds	Stocker-Wörgötter and Hager, 2008
*Peltigera aphthosa*	Mix medium: (8 g/L) Peptone from meat, (8 g/L) Peptone from caseine, (20 g/L) Malt extract, (3 g/L) yeast extract, (5 g/L) Nacl, (40 g/L) Glucose, (15 g/L) Agar		Promote the production of polyketides compounds	Stocker-Wörgötter and Hager, 2008

### MS-based spectrometry as the core technology—Molecular network strategy

In the search for secondary metabolisms, analytical methods must be determined to use for the detection of the compounds (Scherlach and Hertweck, 2009; Tarkka et al., 2009; Palazzotto and Weber, 2018; Manish and Yogesh, 2019). The methods for identification and determining lichen metabolites in the liquid or solid medium are traditionally chemical empirical processes, which include classic spot tests, micro-crystallography, TLC, and HPLC. Kim confirmed the metabolite of *Cladonia rangiferina* by using HPLC and reported that usnic acid could not be found in *C. rangiferina* despite the gene cluster producing usnic acid being observed in the genome (Kim et al., 2021). However, these cheap but not sensitive enough methods will fail when the quantities of metabolites are below the detection limit or when the similar retention time of other metabolites overlaps (Egbert et al., 2022).

Mass spectrometry (MS) is a fast, modern, and simple tool for the structure identification of chemical substances (Wambui et al., 2021), and many lichen compounds and functional groups have been identified using MS (Huneck, 1999). In recent years, several studies focusing on lichen chemistry highlighted the use of a range of hyphenated technology. Mass spectrometry (MS), due to its sensitivity and Nuclear Magnetic Resonance (NMR) spectroscopy coupled with chromatographic techniques, has been recognized as a key technology to study metabolomics (Krug and Muller, 2014). It has been well-demonstrated that liquid chromatography (LC)–MS/MS is considerably more sensitive for the analysis of usnic acid (Cansaran et al., 2006; Sveshnikova et al., 2019; Xu et al., 2022).

Mass spectrometry (MS)/MS-based molecular networking and extensive spectroscopic analyses involving GIAO (Gauge-Independent Atomic Orbital) NMR shift calculation led to the isolation and identification of novel quinoid lichen pigments (Lagarde et al., 2021). However, although MS is the most sensitive and powerful method which detects and elucidates extremely low-abundance metabolites occurring in natural product research, it does not provide any information concerning the spatial and temporal distribution of metabolites. Mass Spectrometry Imaging (MSI) visualizes the production, location, and distribution of metabolites, which is newly used in lichens to visualize the accumulation of various polyketides such as parietin, physodic acid, atranorin, and pinastric acid in different tissues of the lichen and localize the phenolic compounds (Gadea et al., 2020). Desorption electrospray ionization-imaging mass spectrometry (DESI-IMS) realized the spatial distribution of usnic acid in cross-sections of the lichen thallus (Xu et al., 2022).

Some other MS-based metabolomics tools, such as electron ionization-mass spectrometry (EI-MS) (Kai et al., 2017), high performance liquid chromatography-diode array detector-mass spectrometry (HPLC-DAD-MS) (Castro et al., 2017), electron spray ionization-mass spectrometry (HESI-MS/MS) fragmentation patterns (Castro et al., 2017), and liquid chromatography-diode array detector-tandem mass spectrometry (UPLC-PDA-MS/MS) (Kumar et al., 2018), also help to identify various novel lichen metabolites and increase the understanding on a complex biological system.

To facilitate the lichen chemistry, an open-access MS/MS-based library with 250 metabolites known as the Lichen DataBase (LDB) was published by Olivier–Jimenez team. To aid this area of research, the MetaboLights database was generated containing the MS spectra of metabolites. Complementing this, the GNPS platform (CCMSLIB00004751209 to CCMSLIB00004751517) contains the merged spectra of these metabolites within a metadata file. Such a fundamental database empowers research on lichen chemistry by prioritizing novel metabolites (Olivier-Jimenez et al., 2019).

In addition to MS, NMR spectroscopy has also been widely used to determine the structure of organic molecules, which is typically coupled with LC/GC for quantitative analysis of low molecular weight compounds. Metabolite profiles in crude extracts of *Xanthoria elegans* thalli during hydration and dehydration were assessed by using ^31^P- and ^13^C-NMR, and approximately 30 metabolites were identified and quantified (Aubert et al., 2007). The ^1^H-NMR spectra of *Xanthoria parietina* and *Peltigera horizontalis* crude extract displayed lichen-specific features with strong signals and confirmed untargeted analysis on a quantitative basis (Eisenreich et al., 2011). Moreover, NMR demonstrates which atoms are present in neighboring groups. Ultimately, NMR can provide information on how many atoms are present in each of these environments (Altemimi et al., 2017). Other imaging techniques such as Raman microscopic analysis can provide time-resolved information about the distribution of major compounds in lichens (Edwards et al., 1997; Liao et al., 2010; Gadea et al., 2020; Xu et al., 2022). FTIR imaging and Raman microscopy were used to localize the presence of usnic acid in *Cladonia arbuscular, C. Uncialis*, and *C. sulphurina* (Liao et al., 2010).

### Genome mining-based strategy

If it is the common fact that is not easily solved now about the conflict between more lichen natural products by OSMAC from LFF cultures and uncertain or not very well bioactivity compared with those isolated from lichen thallus, genome mining-based strategy will be a more explicit way to discover lichen natural products. With the development of bioinformatics and the applying next-generation sequencing data, there has indeed been more focus on natural product discovery based on genomics (Garima et al., 2022b; Luo et al., 2022). Genome mining has become a powerful tool to discover compounds, identify cryptic biosynthetic gene clusters, characterize the potential biosynthetic pathways, and predict the skeletal structure of the relative products (Liu Q. et al., 2022; Liu T. et al., 2022; Kalra et al., 2023). An increasing understanding of high-quality genome sequencing and genome mining techniques coupled with the introduction of powerful computational toolkits facilitates the process of connecting these gene clusters with key compounds (Li et al., 2016). Comparing the traditional method for the identification of biosynthetic gene clusters by using MS and NMR-based, *in silico* genome mining has become a crucial strategy for the discovery and characterization of gene clusters (Alam et al., 2022). Many web portals contain databases and tools to identify the metabolites by using BLAST, Diamond, and HMMer alignment tools. After uploading the genome to websites, the results of the detection and characterization of secondary metabolites are achieved soon. AntiSMASH (Medema et al., 2011), PRISM (Skinnider et al., 2017), and MIBiG (Kautsar et al., 2020) are representative *in silico* tools for predicting various types of gene clusters, and they were developed to automate biosynthetic gene clusters instead of much manual intervention in genome sequences (Kenshole et al., 2021). Among those three tools, antiSMASH is the largest database of biosynthetic gene cluster analysis, PKS, and non-ribosomal peptide synthase (NRPS) substrate specificity prediction, as well as known and unknown biosynthetic gene clusters comparison (Medema et al., 2011). In addition, antiSMASH was used to predict the molecular structure sequence database. *In silico* genome tool with antiSMASH and BLAST2GO programs investigated the type I-PKS module candidates in nine publicly available LFF genomes (Erken et al., 2021). In addition, rule-based tools such as antiSMASH, ClusterFinder, and RNNs, and machine learning tools have been developed to allow the identification of unknown biosynthetic gene clusters (Cimermancic et al., 2014; Hannigan et al., 2019). However, a much higher false-positive rate than the rule-based tools is the weakness of machine learning-based genome mining tools. Open sources such as Prodigal and automated annotation help reduce false-positive identification. All these softwares are powerful tools that help to make genome mining *in silico* of the interesting LFF.

The genome mining study of a few publicly available LFF genomes suggested the importance of genome mining at the strain level, as it increases the likelihood that researchers discover useful derivatives of known secondary metabolites. An integrated approach utilizing genomics and metabolomics is needed to study the lichen complex systems. Recently, genome mining and comparative genomics strategy were used to assess biosynthetic gene clusters and putative regulators of LFF *Evernia prunastri* and *Pseudevernia furfuracea*. The results showed that the NR-PKS from LFF *Pseudevernia furfuracea* produces depside lecanoric acid, which has never been detected from lichen thallus in nature (Calchera et al., 2019). Genome mining analysis based on a homology searching approach revealed that enzymes of grayanic acid, patulin, and betaenones A-C biosynthesis are encoded by *Cladonia uncialis* genome (Bertrand et al., 2018), and the result corresponds with Shishido et al. (2021). The uptake of advanced analytical techniques and next-generation computational tools brought a breakthrough in lichen chemistry and resulted in the identification of various novel compounds. Moreover, understanding the genetic components leading to the biosynthesis of these metabolites provides an opportunity to exploit their commercial utilization by employing synthetic biology approach.

In LFF, polyketides are the most common class of secondary metabolites. With the help of gene knockout studies, it has been observed that cryptic PKS gene codes for PKS required for the biosynthesis of the representative polyketide orsellinic acid. Polyketides synthesized by three types of multidomain polyketide synthases in the sequential acyl acetyl-polymalonyl pathway are major, structurally diverse classes of natural products (Lin and Qu, 2022; Liu Q. et al., 2022). In the case of bacteria and fungi, PKSs belong to types I and II, while type III is present in higher plants. Despite structural differences, almost all PKSs biosynthesize polyketides via sequential decarboxylative Claisen condensation of acyl-coenzyme A (CoA) precursors and use ketoacyl synthase to catalyze the C–C bond formed during carbon chain assembly, and this process is as similar as fatty acid synthases (Lin and Qu, 2022). From the ecological perspective, these polyketide-based secondary metabolites afford a large amount of cytotoxic or antibiotic compounds to adapt to the competitive living environment. Many of these compounds or their derivatives have emerged as clinically useful drugs or are promising drug candidates. Genetic regulation study of lichen or LFF secondary metabolism is at an early stage, and as time passes and technology advances, more and more research will be covered in this field (Valarmathi et al., 2009; Calchera et al., 2019; Singh et al., 2021). Recently, several LFF PKS genes have been cloned, such as type I NR-PKS gene *XsmPKSI* from *Xanthoria substrigosa* (Hametner and Stocker-Wörgötter, 2015); three new NR-PKS genes such as *UlPKS2, UlPKS4*, and *UlPKS6* from *Usnea longissima* (Wang et al., 2014); and *XsePKS1* from *Xanthoria semiviridis* (Chooi et al., 2008). In addition, some studies of polyketide synthase genes have also focused on phylogenomic analysis (Proctor et al., 2007; Wang et al., 2018; Kealey et al., 2021). The increasing number of phylogenomic analyses shows that a single fungal genome may contain more than one PKS gene, and each species of fungi can produce more than one polyketide or polyketide family (Stocker-Wörgötter, 2008). For example, 12 PKS genes have been identified in *Cladonia grayi* (Shukla et al., 2010). Armaleo et al. (2011) identified a likely orcinol decided PKS and other pathway genes in its metabolic cluster, and it was the first genetic evidence for a complete depside/depsidone biosynthetic pathway. Experimental data that seven complete non-reducing and nine highly-reducing PKS genes indicated *Nppks7* was a new PKS that participated in usnic acid biosynthesis in LFF *Nephromopsis pallescens* (Wang et al., 2018).

The complex lichen biology as filamentous fungi, transcriptionally silent, and trace expression make an artificial synthesis of interesting secondary metabolites difficult (Harvey et al., 2018). Heterologous expression of biosynthetic gene clusters in a non-natural host or model system expedites natural product discovery, elucidation, and mass production. The apposite choice of host is one of the keys to successful heterologous expression. Due to many advantages, such as fast growth, high cell density, low cost, simple cultivation medium, fast transformation procedure, and ability to process and correctly splice introns, *Saccharomyces cerevisiae* (Kealey et al., 2021), *Fusarium venenatum* (Sinnemann et al., 2000), *Aspergillus nidulans* (Sinnemann et al., 2000), *A. niger* (Sinnemann et al., 2000), *A. oryzae* (Gagunashvili et al., 2009), and *Neurospora crassa* are the experimentally well-developed strains and are considered as the potential hosts for the expression of lichen DNA (Qiao et al., 2019). In other expression systems, to avoid the influence of the surrogate host's metabolism on heterologous biosynthesis, *Ascochyta rabiei*, chosen as the host, is a genetically tractable, wild-type plant-pathogenic fungus without the biosynthetic gene cluster of phytotoxic solanapyrones (Kim et al., 2021).

Using PKS genes as a heterologous expression of genes for filamentous fungal secondary metabolites has been widely reported (Gressler et al., 2011; Sakai et al., 2012; Qian et al., 2020). Although PKS genes of lichen and filamentous fungi showed the greatest homology, only a few PKS genes have been isolated and characterized functionally from lichen or LFF. As the first PKS gene from *Solorina crocea* LFF, *PyrG* encoding decarboxylase was functionally expressed under its own promoter in *A. nidulans* (Sinnemann et al., 2000). The result indicated that a heterologous expression system is a useful tool for the functional characterization of genes. Another example is that two pairs of degenerated primers have been used to locate and clone PKS genes containing a CMeT domain from *Xanthoparmelia semiviridis* (Chooi et al., 2008). Early functional research of lichen PKS genes mainly focused on symbiosis, physiology, and biochemistry because all the studies that attempted to express the PKS gene of unique lichen compounds failed (Chooi et al., 2008). Until recently, *de novo* biosynthetic PKS genes of atranorin and lecanoric acid have been successfully heterologously expressed (Kealey et al., 2021; Kim et al., 2021). Atranorin is one of the most concerned lichen compounds. The results from lichens such as *Cladonia, Stereocaulon alpinum* (Kim et al., 2021), and *Bacidia rubella* (Gerasimova et al., 2022) revealed that the PKS23 gene (*atr1*), a cytochrome P450 gene (*art2*) for oxidation, an O-methyltransferase (OMT) gene (*atr3*), and transporter gene (*atr4*) were involved in producing atranorin.

## Conclusion and future perspectives

Lichen secondary metabolites are of major interest due to their applicability as therapeutic agents. However, a special way of symbiosis, extreme living environment, and slow growth of lichen limit the constant need for lichen compounds in industry and pharmacy. The analysis of genome sequence revealed that there exist silent biosynthetic gene clusters, which are usually not expressed until being activated, leading to the discovery of lichen compounds much inadequate. OSMAC strategy is a powerful and mature method for enhancing the chemodiversity of LFF natural compounds, under which new drugs could be obtained by manipulating nutritional or environmental factors of fermentation to activate silent gene clusters. However, sometimes, the results of the OSMAC strategy are not very satisfying because it has a limited capacity to mimic the complexities of natural environmental changes. MS-based molecular network strategy further facilitates lichen chemistry, especially linked to a series of databases such as LDB and MetaboLights. Genome- and bioinformatics-based genome mining strategy not only makes up for the difficulties and shortcomings of the OSMAC strategy but also strongly pushes the identification of biosynthetic gene clusters and increases the rate of discovery of new products. Genome mining strategy, covering several different usage cases in animal, plant, and microbe, shows diverse ways, in which genomic data can be used to uncover new secondary metabolites, improves our understanding of their biosynthesis, and uncovers long-term biosynthetic mysteries, but for lichen, it is just the beginning. Nowadays, various strategies for inducing the expression of silent biosynthetic gene clusters have been developed. However, each strategy has its own limitations, and no strategy could be universal for all strains. Furthermore, significant advances are needed in terms of the enrichment of the database for lichen metabolites together with the general standardization of different generations of data. A combination of OSMAC, molecular network, and genome mining-based strategies will be greatly helpful to predict the biosynthesis and accumulation of specific natural products, discover numerous novel secondary metabolites with a range of attractive bioactivities, and pursue the establishment and maintenance of the lichen symbiosis.

## Author contributions

XW: conceptualization. MR: writing—original draft preparation. SJ, YW, XP, FP, and XW: writing—reviewing and editing. All authors read and approved the manuscript.
